# Comparison of smoking prevalence in Canada before and after nicotine vaping product access using the SimSmoke model

**DOI:** 10.17269/s41997-023-00792-3

**Published:** 2023-08-04

**Authors:** David T. Levy, Christopher J. Cadham, Zhe Yuan, Yameng Li, Shannon Gravely, K. Michael Cummings

**Affiliations:** 1 Lombardi Comprehensive Cancer Center, Georgetown University, Washington, DC USA; 2 Department of Health Management and Policy, University of Michigan, Ann Arbor, MI USA; 3 Department of Psychology, University of Waterloo, Waterloo, ON Canada; 4 Department of Psychiatry & Behavioral Science, Medical University of South Carolina, Charleston, SC USA

**Keywords:** E-cigarettes, ENDS, Smoking, Simulation model, Canada, Cigarettes électroniques, dispositifs électroniques d’administration de nicotine, tabagisme, modèle de simulation, Canada

## Abstract

**Objectives:**

The public health impact of nicotine vaping products (NVPs) is subject to complex transitions between NVP and cigarette use. To circumvent the data limitations and parameter instability challenges in modeling transitions, we indirectly estimate NVPs’ impact on smoking prevalence and resulting smoking-attributable deaths using the *SimSmoke* simulation model.

**Methods:**

*Canada SimSmoke* uses age- and sex-specific data on Canadian population, smoking prevalence and tobacco control policies. The model incorporates the impact of cigarette-oriented policies on smoking prevalence but not the explicit contribution of NVPs. The model was calibrated from 1999 to 2012, thereby projecting smoking prevalence before NVPs were widely used in Canada. The NVP impact on smoking prevalence is inferred by comparing projected 2012–2020 smoking trends absent NVPs to corresponding trends from two Canadian national surveys. We further distinguish impacts before and after NVPs became regulated in 2018 and more available.

**Results:**

Comparing 2012–2020 survey data of post-NVP to SimSmoke projected smoking prevalence trends, one survey indicated an NVP-related relative reduction of 15% (15%) for males (females) age 15+, but 32% (52%) for those ages 15–24. The other survey indicated a 14% (19%) NVP-related smoking reduction for ages 18+, but 42% (53%) for persons ages 18–24. Much of the gain occurred since Canada relaxed NVP restrictions. NVP-related 2012–2020 smoking reductions yielded 100,000 smoking-attributable deaths averted from 2012 to 2060.

**Conclusion:**

Smoking prevalence in Canada, especially among younger adults, declined more rapidly once NVPs became readily available. The emergence of NVPs into the Canadian marketplace has not slowed the decline in smoking.

**Supplementary Information:**

The online version contains supplementary material available at 10.17269/s41997-023-00792-3.

## Résumé

**Objectifs:**

L’effet des produits de vapotage avec nicotine (PVN) sur la santé publique dépend des transitions complexes entre l’usage des PVN et l’usage de la cigarette. Pour contourner les problèmes du manque de données et de l’instabilité des paramètres dans la modélisation de ces transitions, nous avons estimé indirectement l’effet des PVN sur la prévalence du tabagisme et sur les décès attribuables au tabagisme qui en résultent à l’aide du modèle de simulation *SimSmoke*.

**Méthode:**

Le modèle *SimSmoke* pour le Canada utilise des données par âge et par sexe sur la population canadienne, la prévalence du tabagisme et les politiques antitabac. Il intègre l’effet des politiques axées sur la cigarette sur la prévalence du tabagisme, mais pas explicitement l’apport des PVN. Ce modèle a été étalonné de 1999 à 2012; il prédit donc la prévalence du tabagisme avant l’utilisation des PVN à grande échelle au Canada. Nous avons déduit l’effet des PVN sur la prévalence du tabagisme en comparant les tendances de consommation de tabac projetées pour 2012–2020 sans PVN aux tendances correspondantes de deux enquêtes nationales canadiennes. Nous établissons aussi une autre distinction entre les effets avant et après la réglementation des PVN en 2018 et leur plus grande disponibilité.

**Résultats:**

Si l’on compare les données d’enquête de 2012–2020 post-PVN aux tendances de prévalence du tabagisme projetées par *SimSmoke*, une enquête fait état d’une baisse de 15 % (15 %) liée aux PVN chez les hommes (femmes) de 15 ans et plus, mais de 32 % (52 %) chez les 15 à 24 ans. L’autre enquête fait état d’une baisse du tabagisme de 14 % (19 %) liée aux PVN chez les 18 ans et plus, mais de 42 % (53 %) chez les 18 à 24 ans. Une grande partie de ce gain s’est produit depuis que le Canada a assoupli ses restrictions sur les PVN. Les baisses du tabagisme liées aux PVN survenues entre 2012 et 2020 donnent 100 000 décès attribuables au tabagisme évités entre 2012 et 2060.

**Conclusion:**

La prévalence du tabagisme au Canada, surtout chez les jeunes adultes, a baissé plus rapidement lorsque les PVN sont devenus facilement accessibles. L’émergence des PVN sur le marché canadien n’a pas ralenti la baisse du tabagisme.

## Introduction

Canada has been one of the leading countries in implementing strong demand-reducing tobacco control policies. In 2001, Canada was the first country to introduce large pictorial health warnings on cigarette packs, and has since adopted robust policies including bans on indoor smoking, advertising and menthol cigarettes, as well as high cigarette taxes (World Health Organization (WHO), 2021), Over the past five decades, Canada has witnessed a remarkable reduction in cigarette smoking prevalence. Approximately half of Canadians smoked cigarettes in 1965, compared to just 15.1% in 2017 (WHO, 2021; Reid et al., 2019). Nevertheless, smoking is responsible for 47,000 premature deaths each year and is the leading preventable cause of death in Canada (Statistics Canada, 2022). More action is needed in order to meet the Canadian Government’s tobacco endgame goal of less than 5% smoking prevalence by 2035 (Tobacco Endgame Cabinet, 2019).

While smoking is declining, the Canadian nicotine market has evolved with the emergence of non-combustible nicotine vaping products (NVPs, also commonly known as e-cigarettes). Before 2018, vaping products containing nicotine were prohibited from being sold in Canada unless licensed by Health Canada; however, this restriction was not widely enforced. NVPs were sold in retail outlets including vape shops and online despite the fact that no manufacturers had received licenses to legally sell NVPs (Hammond et al., 2015; Reid et al., 2015). By 2017, past-30-day use of NVPs increased to 3% overall and 6% among those aged 15–24 (Statistics Canada, 2019). In May 2018, the Tobacco and Vaping Products Act (TVPA) was enacted, allowing NVPs to be sold in retail stores (e.g., convenience stores, gas stations, vape shops) without a license, but with a required text health warning and restrictions on marketing (Hammond et al., 2020; WHO, 2021). By 2019, past-30-day NVP u**s**e was 5% among those aged 15+, but use increased most sharply among youth and young adults (Government of Canada, 2020).

The availability of NVPs represents a new challenge for tobacco control. Access to non-medicinally marketed nicotine products offers those who smoke cigarettes an opportunity to substitute a lower-risk NVP for their cigarettes (McNeill et al., 2022; National Academy of Sciences, Engineering and Medicine (NASEM), 2018). Some studies find that NVP use motivates quit attempts (Kasza et al., 2022) and successful cessation (Hartmann-Boyce et al., 2021), and may reduce cigarette uptake in those predisposed to smoke (Levy et al., 2019a; Meza et al., 2020). However, other studies suggest that NVP use may lead non-smoking youth to initiate smoking (Soneji et al., 2017; Watkins et al., 2018), and some smokers may be less likely to quit (Miller et al., 2020, NASEM, 2018) and more likely to relapse (Dai & Leventhal, 2019). The public health impact of NVPs depends primarily on their health risks and impact on cigarette smoking behaviour (Levy et al., 2017). However, NVP risks are uncertain (McNeill et al., 2022; NASEM, 2018) and transitions to and from NVP and cigarette use change due to product evolution in nicotine delivery, attractiveness, and cost (Abrams, 2014), all of which lead to unstable patterns of NVP use and transitions at the population level.

When direct evidence is limited, simulation can be used as a virtual laboratory to synthesize disparate sources of data to examine the public health impact of patterns of nicotine product use. Currently, there is limited evidence on the role NVPs may have played in recent declines in cigarette smoking in Canada. Taking advantage of Canadian time-series data from two nationally representative surveys, this study indirectly estimated NVPs’ impact on smoking prevalence and resulting smoking-attributable deaths using the Canada *SimSmoke* simulation model.

Because of the lack of reliable data to explicitly model the transitions between NVP and cigarette use, an indirect method was applied that was previously used in England (Levy et al., 2021a) and the United States (Levy et al., 2021b). The approach described herein compares projections from a *No-NVP* counterfactual scenario to actual smoking rates with NVPs in the marketplace. The counterfactual scenario is developed using the well-established and validated *SimSmoke* tobacco control simulation model (Levy et al., 2016a), which controls for cigarette-oriented policies, but does not incorporate the explicit contribution of NVPs. The analyses represent an extension of our previous applications to consider the impact of reduced restrictions on cigarette smoking prevalence reductions before and after NVPs became allowable for sale in the post-TVPA period.

## Methods

*SimSmoke* applies a first-order Markov process to population and smoking initiation and cessation, and incorporates the effect of tobacco control policies to project smoking prevalence and smoking-attributable deaths. The model has been shown to predict well (Levy et al., 2008, 2012, 2014a, b, 2016a). The *Canada SimSmoke* model begins in the year 1999, a period of stabilization after tobacco control policy changes but before major policy changes in 2001 (Supplement 1 Figure), and thus a suitable period to calibrate initial smoking trends. The model is briefly described below and more fully described in a Supplemental Report.

### Population and smoking prevalence

The population evolves through births, deaths, and net international migration up to 2060. Census population estimates for the period 1999–2018 (Statistics Canada, 2020b) with projections for the period 2019–2060 (Statistics Canada, 2020b) were obtained by age and sex from Statistics Canada. Births are based on population estimates.

*Canada SimSmoke* distinguishes the 1999 baseline population by age- and sex-specific never, current, and former smoking prevalence using the 1999 Canadian Tobacco Use Monitoring Survey (Statistics Canada, 2013). Three questions were considered: “At the present time do you smoke cigarettes every day, occasionally, or not at all?”; “Have you ever smoked at least 100 cigarettes in your life?”; and “When did you stop smoking?” Based on the survey questions, current smokers were defined as those who have smoked at least 100 cigarettes life-time, and currently smoke either every day or some days. Former smokers meet the 100-cigarettes lifetime criterion but do not currently smoke, and are distinguished by years quit.

Due to empirical challenges in measuring initiation and quitting and to ensure stability and internal consistency of the model, initiation is modelled net of quitting and relapse, measured by the difference between smoking prevalence at a given age and the previous age relative to never-smoking prevalence at the previous age. Net initiation occurs through age 20 for males and age 21 for females, the ages at which smoking prevalence stopped increasing. Cessation is modeled after age 20 for males and 21 for females, measured as the ratio of former smokers who quit in the last year to smoking prevalence in the previous year. Lacking Canadian data, US relapse rates by age, sex, and years quit (U.S. DHHS, 1990; Gilpin et al., 1997) were applied to capture relapse from former to current smokers. Smoking relapse in Canada has been found to be related to similar factors as in the US (Yong et al., 2018).

Smoking-attributable deaths (SADs) are estimated for current and former smokers. Relative risks of current and former smokers are based on US risks, estimated from the Cancer Prevention Study II (Burns et al., 1997; U.S. DHHS, 1989). Relative risks are combined with Canadian smoking prevalence rates to estimate death rates for never, current, and former smokers (CDC, 2000). The number of smokers at each age is multiplied by age- and sex-specific excess smoker risks (current minus never smokers death rate) to obtain smoker excess deaths. The same method is used for former smokers. Deaths are then summed over current and former smokers of all ages to obtain total SADs.

### Tobacco control policy impacts

*Canada SimSmoke* begins with the level of tobacco control policies in Canada in 1999 and then incorporates any changes through 2020. Policy effect sizes (PES, PES<0) are generally applied as an immediate reduction in smoking prevalence (1+PES) in the first year, and applied to the initiation rate as (1+PES) and the cessation rate as (1-PES) in future years. The effects of different policies are generally multiplicatively applied, i.e., (1+PES_i_)*(1+PES_j_) for policy *i* and *j*, implying that policy impacts are independent but the absolute impact is reduced when another policy is simultaneously implemented. Policy effect sizes are based on published literature reviews, Canada-specific studies and the advice of experts. Policies and their effect sizes are provided in Table 1.

**Table 1:** Tobacco control policies, specifications and effect sizes applied in Canada SimSmoke

Policy	Description	Policy effect size
Cigarette Excise Taxes		
Cigarette price/tax	The effect of taxes is directly incorporated through the average price after tax. The price elasticity is used to convert the price changes (%) into effect sizes	Elasticities: -0.6 for ages 15–20, -0.2 for ages 21–34, -0.1 for ages 35–64, -0.2 for ages 65+
Smoke-free Air Laws		
Worksite smoking ban	Ban in all indoor worksites, with strong enforcement of laws (reduced by 1/3 if allowed in ventilated areas and by 2/3 if allowed in common areas)	-6% prevalence and initiation, +6% cessation
Restaurant smoking ban	Ban in all indoor restaurants (scaled for lower coverage), with strong enforcement of laws	-2% prevalence and initiation, +2% cessation
Pubs and bars smoking ban	Ban in all indoor pubs and bars (scaled for lower coverage), with strong enforcement of laws	-1% prevalence and initiation, +1% cessation
Other place bans	Ban in 3 out of 4 government buildings (scaled for lower coverage), retail stores, public transportation, and elevators, with strong enforcement of laws	-1% prevalence and initiation, +1% cessation
Enforcement and publicity	Government agency enforces the laws and publicity via tobacco control campaigns	Effects reduced 50% absent publicity and enforcement
Marketing Restrictions		
Comprehensive marketing ban	Ban on all forms of direct advertising and indirect marketing	-5% prevalence,
		-8% initiation,
		+4% cessation
Moderate advertising ban	Ban on broadcast media, newspapers and billboards marketing and at least some indirect marketing (sponsorship, branding, giveaways)	-3% prevalence,
		-4% initiation,
		+2% cessation
Minimal advertising ban	Ban on broadcast media advertising	-1% prevalence and -1% initiation only
Enforcement	Government agency enforces the laws	50% scaled to enforcement
Retail point-of-sale (POS) restriction	Restrict the visibility and accessibility of tobacco products at the point of sale	-12% initiation,
		+10% cessation
Health Warnings		
High health warnings	Labels are large, bold and graphic, and cover at least 50% of package	-4% prevalence,
		-6% initiation,
		+10% cessation
Moderate health warnings	Laws cover at least 30% of package, not bold or graphic	-2% prevalence,
		-2% initiation,
		+4% cessation
Low health warnings	Laws cover less than 30% of package, not bold or graphic	-1% prevalence,
		-1% initiation,
		+2% cessation
Additional impact of plain packaging w/ strong warnings	The outside of the package is drab, with brand and variant names appearing once on the front, top and bottom surfaces, and no inserts	-2% prevalence,
		-2% initiation,
		+2% cessation
Media Campaigns		
High-level media campaign	Campaign publicized heavily with state and local programs with strong funding (>$0.50 USD per capita)	-6.5% prevalence and initiation, +6.5% cessation
Moderate-level media campaign	Campaign publicized with funding of at least $0.10 USD per capita	-3.25% prevalence and initiation, +3.25% cessation
Low-level media campaign	Campaign publicized only sporadically with minimal funding (<$0.10 USD per capita)	-1.63% prevalence and initiation, +1.63% cessation
Cessation Treatment Policies		
Availability of pharmacotherapies	Legality of nicotine replacement therapy and/or Bupropion and Varenicline	-1% prevalence,
		+4% cessation
Cessation treatment financial coverage	Payments to cover pharmacotherapy and behavioural cessation treatment with high publicity (effect size reduced by 12.5% with moderate publicity and 18.75% with low publicity)	-2.25% prevalence,
		+8% cessation
Quit line	Three quit-line types: passive, proactive and active with follow-up (effect size reduced by 1/3 if quit line is proactive only, reduced by 2/3 if quit line passive only)	-1% prevalence,
		+6% cessation
Brief interventions	Advice by health care provider to quit and methods provided	-1% prevalence,
		+6% cessation
All cessation policies combined	Complete availability and reimbursement of pharmaco- and behavioural treatments, quit lines, and brief interventions	-5.68% prevalence,
		+29.40% cessation
Youth Access Policies		
Strong enforcement and well publicized	Compliance checks are conducted 4 times per year per outlet, penalties are potent and enforced with heavy publicity	-16% initiation and prevalence for ages 16–17 and -24% for ages 10–15
Moderate enforcement with some publicity	Compliance checks are conducted regularly, penalties are potent, and publicity and merchant training are included	-8% initiation and prevalence for ages 16–17 and -12% for ages 10–15
Low enforcement	Compliance checks are conducted sporadically, penalties are weak	-2% initiation and prevalence for ages 16–17 and -3% for ages 10–15
Menthol Bans		
Population coverage	Menthol in cigarettes banned federally in 2017 and in some provinces before 2017	-0.5% prevalence, -2% initiation

Unless otherwise indicated, the policy effect sizes are in terms of the reduction in prevalence during the first year, and the reduction in initiation and increase in quit rates during future years that the policy is in effect

*SimSmoke* models cigarette tax changes. Changes in price are translated into changes in smoking prevalence using prevalence elasticities from demand studies (Chaloupka et al., 2000). Canadian studies (Azagba et al., 2015; Gagné, 2021) obtain elasticity estimates consistent with those from other high-income nations (Chaloupka et al., 2011). The model uses CPI-adjusted prices for 1999–2019 from Gagné (Gagné, 2021), measured by the annual average of provincial retail cigarette prices. For 2020, the 2019 cigarette price was scaled by the ratio of 2020 to 2019 Canadian cigarette prices from the Economist Intelligence Unit (The Economist Intelligence (EIU), 2021).

Smoke-free air laws include worksites, restaurants, pubs and bars, and other public places. Studies find effect sizes for Canada (Hammond et al., 2004) consistent with other countries (Levy et al., 2018a). Studies (Hammond et al., 2004; Reid et al., 2016) found that 60% of public places were well covered in 1999 and most provinces were fully covered by 2009. The impact of smoke-free air laws depends on enforcement, rated as high (9 on a 10-point scale) in all years based on Zhang et al. (2010) and World Health Organization Reports (WHO, 2015, 2017, 2021).

Marketing restrictions also depend on enforcement and are classified as minimal, moderate, and complete. The Tobacco Products Control Act of 1988 and Tobacco Act of 1997 banned most forms of advertising (Reid et al., 2016). Bill C-32 (Parliament of Canada, 2009) in 2009 removed tobacco advertising in newspapers and limited magazines and sponsorship and branding (WHO, 2015, 2017). A moderate level is assigned in 1999, increasing to 25% moderate and 75% complete ban in 2009. Based on an ITC Report (ITC Project, 2013), a level 8 (of 10) is assigned for enforcement in 1999–2008, increasing to 9 from 2009 onward based on WHO Reports (WHO, 2015, 2017, 2021). *SimSmoke* separately incorporates retail point-of-sale (POS) display restrictions as implemented by each province (Levy et al., 2015), culminating in nationwide implementation by January, 2010 (Reid et al., 2016).

For health warnings on cigarette packages*, SimSmoke* distinguishes low, moderate, and strong with additional impacts for plain packaging. Effect sizes are based on a review (Levy et al., 2016b), which included Canadian studies (Azagba et al., 2020; Hammond, 2011). Health warnings were increased to pictorial, rotating and covering 50% of both principal sides in 2011 and were required to cover 75% of both sides with a toll-free quitline number in 2012 (Reid et al., 2016). A moderate level is assigned through 2000, increasing to 50% moderate and 50% strong in 2001 and 100% strong onward from 2012. Effect sizes for plain packaging, implemented in 2019, are based on McNeill et al. (2017).

Tobacco control media campaigns are classified as high, moderate and low based on tobacco control expenditures, most of which is mass educational programs conducted through media and local programs. Based on ITC Project (2013) and WHO (2015, 2017, 2021), a low level is assigned for 1999–2002, increasing to a moderate level in 2003–2020.

Cessation treatment policies include financial coverage of pharmacotherapy and behavioural treatments, quitlines, and brief interventions. Effect sizes for Canada (Cunningham et al., 2016; Stich et al., 2021; Voci et al., 2016) are consistent with Levy et al. (2010). Pharmacotherapies were available since 1999 and partially covered by insurance starting in 2003 with coverage increasing by 2011 (Dubray & Schwartz, 2010). NRT and behavioural counseling were at least partially cost-covered throughout much of Canada by 2008. In 2000, a national toll-free quitline became available and physicians were recommended to provide brief interventions (Canadian Cancer Society (CCS), 2010). Canadian studies (Cunningham et al., 2011; Leatherdale & Shields, 2009) find that 40–50% of physicians provide brief interventions, but fewer provide follow-up. A value of 25% is assigned for brief interventions in 1999 increasing to 35% in 2001.

Youth access considers the effect of retail compliance with minimum purchase age laws (Levy et al., 2001). The 1997 Tobacco Act prohibits the sale of tobacco products to persons below age 19. A Report (Government of Canada, 2016) found non-compliance rates of 30% from 1999 to 2003 falling to 20% from 2004 to 2009, as recently confirmed (Minaker et al., 2015). Enforcement is set to low from 1999 to 2003 and moderate since 2003.

After earlier provincial bans, a federal ban on menthol in cigarettes and little cigars was implemented in October 2017. Recent studies (Cadham et al., 2020; Chaiton et al., 2021) indicate 7–8% relative reductions in smoking prevalence. Using pre-ban menthol smoking rates (Statistics Canada, 2019), an initial 0.5% reduction in smoking prevalence is applied with an ongoing 2% reduction in initiation for Canada, weighted by the percent of population in covered provinces each year prior to 2017.

### Estimating the impact of NVPs

The model projects smoking prevalence and SADs by age and sex for each year. Smoking prevalence estimates are from CTUMS through 2012 (Statistics Canada, 2013), from the updated CTADS (Canada Tobacco and Drug Survey) in 2013–2017 (Statistics Canada, 2019), and CTNS (Canada Tobacco and Nicotine Survey) in 2019–2020 (Government of Canada, 2020). The three surveys are sequentially applied, referenced herein as CTUMS. Smoking prevalence estimates are also generated from the larger 2001–2020 CCHS (Statistics Canada, 2020a). Smoking prevalence was calibrated against smoking prevalence estimates by age and sex from 1999–2012 CTUMS and 2001–2012 CCHS, thereby providing pre-NVP trends. Data after 2012 were used to gauge potential post-NVP impacts.

*Canada SimSmoke* projections do not account for any contribution of NVPs and thus provide the No-NVP counterfactual from 2012 to 2020, the NVP period. The impact of NVPs on smoking is inferred by comparing *SimSmoke*’s No-NVP projected smoking prevalence by age and sex to actual smoking prevalence from the CTUMS and CCHS surveys over the period 2012–2020. The No-NVP counterfactual *SimSmoke* projected relative change in smoking prevalence between 2012 and 2020 is subtracted from the corresponding relative change estimated individually from both CTUMS and CCHS. Separate yearly NVP adjustments are calculated as [(1-*SimSmoke* 2012–2020 smoking relative reduction)^1/8^ - (1-Survey 2012–2020 smoking relative reduction)^1/8^]. These age- and sex-specific NVP-related annual smoking reductions are added back to never smokers during ages of smoking initiation and to former smokers after those initiation ages to estimate the NVP-adjusted smoking prevalence.

Relative changes in the NVP-adjusted smoking prevalence are subtracted from the No-NVP relative reduction to gauge the potential impact of NVPs on smoking prevalence. Uncertainty is gauged by 95% confidence intervals (CIs) of the 2020 survey estimates [e.g., upper bound of the relative reduction = (upper bound of 2020 prevalence-2012 prevalence)/2012 prevalence].

Since NVPs became legally available through the 2018 TVPA, the post-TVPA (2018–2020) were compared to the pre-TVPA (2013–2017) NVP-related relative reductions for CTUMS. However, since CCHS is only available for 2018, the pre- and post-TVPA periods are 2012–2018 and 2018–2020.

To obtain the inferred health impact of NVPs, SADs (from current and former smokers) under the No-NVP and NVP-adjusted scenarios were first separately estimated. The potential impact of 2012–2020 NVP use was estimated as the difference between NVP-adjusted and No-NVP projected SADs over the period 2012–2060.

## Results

### Calibration of smoking prevalence estimates over the pre-VP period (1999–2012)

The initial *SimSmoke* smoking prevalence was calibrated to projections of each survey’s estimates over the period 1999–2012. Initiation rates were reduced for males ages 15–20 and females ages 15–21. Cessation rates were reduced for males below age 45 and at ages 65+ and for females at ages 20–45, and were increased for males ages 45–64 and females ages 45–64.

From 1999 to 2012, the calibrated model predicts a 32.3% (39.0%) relative reduction in male (female) smoking prevalence for ages 15+ compared with a 33.9% (41.5%) CTUMS reduction. From 2001 to 2012, the model predicts a 30.1% (35.0%) relative reduction in male (female) smoking prevalence for ages 18+ compared with an 18.8% (26.7%) CCHS reduction. The differences in the CCHS and CTUMS calibrations may reflect different base-year comparisons (1999 CTUMS and 2001 CCHS) and smoking prevalence measures. As shown in Supplement 2 Table, the model generally calibrated well by age and sex to 2020 levels and 2012–2020 relative reductions.

### Impact of NVPs on smoking prevalence relative to a No-NVP scenario, 2012–2020

**Fig. 1:**
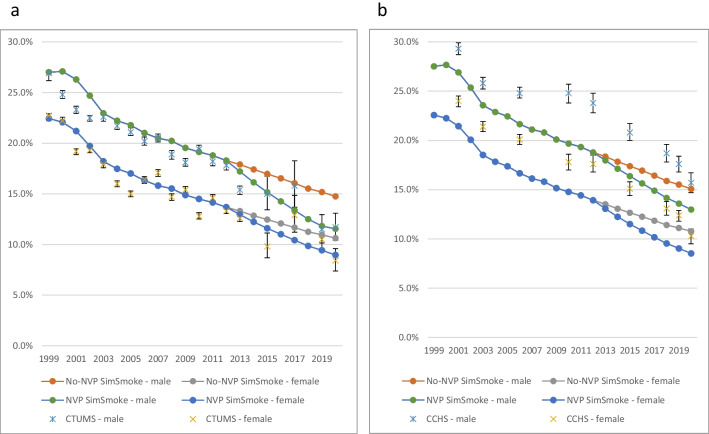
**a** Adult smoking prevalence, No-NVP SimSmoke, CTUMS-adjusted NVP SimSmoke, CTUMS, 1999–2020. **b** Adult smoking prevalence, No-NVP SimSmoke, CCHS-adjusted NVP SimSmoke, and CCHS, 1999–2020

The yearly levels and projected relative reductions in smoking prevalence from the No-NVP *SimSmoke* (counterfactual) and the implied NVP-related reductions from CTUMS and CCHS in 2012–2020 are shown in Tables 2 and 3. Figure 1a and b. shows the CTUMS- and CCHS-adjusted projected No-NVP and NVP smoking prevalence and CTUMS and CCHS estimates through 2020 for both sexes aged 15+.

**Table 2:** Smoking prevalence, No-NVP SimSmoke vs. CTUMS/CTADS/CTNS estimation of the implicit NVP effect, males and females, 2012–2020

Ages	Sources	2012	2017	2020	Relative reduction, 2012–2020	Difference SimSmoke vs Survey, 2012–2020	Annual relative reduction, 2012–2020	Annual relative reduction, 2012–2017	Annual relative reduction, 2017–2020
Males									
15+	SimSmoke	18.3%	16.1%	14.8%	19.3%				
	CTUMS 95% CI	17.8% 17.4%,18.2%	15.8% 13.7%,18.3%	11.7% 10.4%,13.1%	34.3% 26.4%,41.6%	14.9% 7.1%,22.2%	2.5% 1.1%,3.8%	-0.2% -3.1%,2.5%	6.8% 3.3%,10.2%
15–24	SimSmoke	16.0%	13.5%	12.5%	21.8%				
	CTUMS 95% CI	15.7% 15.3%,16.2%	12.3% 10.4%,14.4%	7.3% 5.8%,9.2%	53.5% 41.4%,63.1%	31.7% 19.6%,41.3%	6.1% 3.4%,8.7%	1.5% -1.6%,4.5%	13.4% 6.7%,19.6%
25–44	SimSmoke	24.1%	21.3%	19.1%	20.7%				
	CTUMS 95% CI	22.9% 22.0%,23.8%	17.7% 13.5%,22.9%	12.1% 9.6%,15.3%	47.2% 33.2%,58.1%	26.5% 12.5%,37.4%	4.8% 2.1%,7.4%	2.6% -2.4%,7.6%	8.4% 1.2%,14.9%
45–64	SimSmoke	18.4%	16.7%	15.9%	13.9%				
	CTUMS 95% CI	18.1% 17.4%,18.8%	20.1% 16.4%,24.5%	12.6% 10.4%,15.2%	30.4% 16.0%,42.5%	16.5% 2.1%,28.7%	2.6% 0.3%,4.8%	-4.1% -8.2%,0.1%	12.8% 7.3%,18.1%
65+	SimSmoke	8.2%	7.5%	7.1%	12.5%				
	CTUMS 95% CI	8.4% 7.7%,9.1%	7.6% 4.5%,12.8%	12.5% 10.1%,15.5%	-48.8% -84.5%,-20.2%	-61.3% -97.1%,-32.8%	-6.8% -9.6%,-4.0%	0.2% -10.5%,10.1%	-19.5% -28.3%,-11.4%
Females									
15+	SimSmoke	13.7%	11.7%	10.6%	22.3%				
	CTUMS 95% CI	13.3% 13.1%,13.6%	12.9% 11.2%,14.8%	8.4% 7.4%,9.6%	36.8% 27.8%,44.4%	14.5% 5.5%,22.0%	2.5% 0.9%,4.0%	-2.5% -5.4%,0.2%	10.3% 6.3%,13.8%
15–24	SimSmoke	12.1%	10.1%	9.3%	23.4%				
	CTUMS 95% CI	11.8% 11.4%,12.2%	6.4% 5.1%,7.9%	2.9% 1.9%,4.2%	75.4% 64.4%,83.9%	52.0% 41.0%,60.5%	12.8% 8.8%,17.1%	7.9% 4.2%,11.7%	20.4% 10.4%,30.5%
25–44	SimSmoke	15.8%	12.4%	10.6%	33.0%				
	CTUMS 95% CI	15.2% 14.6%,15.9%	17.5% 13.6%,22.3%	8.4% 6.5%,10.8%	44.7% 28.9%,57.2%	11.7% -4.0%,24.2%	2.3% -0.7%,5.2%	-7.6% -12.6%,-2.5%	16.5% 9.7%,22.9%
45–64	SimSmoke	14.5%	12.8%	11.7%	19.3%				
	CTUMS 95% CI	14.4% 13.9%,15.0%	14.1% 11.3%,17.4%	10.9% 8.9%,13.2%	24.3% 8.3%,38.2%	5.0% -11.0%,18.9%	0.8% -1.6%,3.2%	-2.0% -6.4%,2.3%	5.2% -0.8%,11.2%
65+	SimSmoke	10.0%	9.9%	10.1%	-0.4%				
	CTUMS 95% CI	9.3% 8.8%,9.8%	8.4% 5.7%,12.2%	8.4% 6.6%,10.7%	9.7% -15.1%,29.0%	10.1% -14.6%,29.4%	1.3% -1.7%,4.2%	1.9% -5.7%,9.2%	0.4% -8.0%,8.1%

1. Relative reductions are measured by the relative decrease within a certain period, e.g., prevalence reduces by 25% from 20% in 2012 to 15% in 2019

2. Differences between SimSmoke model projections and survey estimates are measured by the relative reductions within the specified time period

3. Annual reductions are the average annual relative reduction when transforming the relative reduction within the specified time period evenly to the between years, e.g., the annual reduction from 20% in 2012 to 15% in 2020 is 1-(15%/20%)^[1/(2020-2012)]^ = 3.5%

4. Annual differences are measured by the difference between the annual reduction in SimSmoke projections and survey estimates within the specified time period

**Table 3:** Smoking prevalence, No-NVP SimSmoke vs. CCHS estimation of the implicit NVP effect, males and females, 2012–2020

Ages	Sources	2012	2018	2020	Relative reduction, 2012–2020	Difference SimSmoke vs survey, 2012–2020	Annual relative reduction, 2012–2020	Annual relative reduction, 2012–2018	Annual relative reduction, 2018–2020
Males									
18+	SimSmoke	18.8%	15.9%	15.1%	19.7%				
	CCHS 95% CI	23.8% 22.8%,24.8%	18.7% 17.8%,19.6%	15.7% 14.7%,16.7%	34.0% 29.8%,38.2%	14.3% 10.1%,18.5%	2.4% 1.6%,3.1%	1.2% 0.4%,2.0%	5.8% 3.0%,8.8%
18–24	SimSmoke	19.0%	15.1%	14.6%	23.1%				
	CCHS 95% CI	27.2% 24.0%,30.3%	18.5% 15.7%,21.3%	9.5% 6.8%,12.2%	65.1% 55.1%,75.0%	42.0% 32.0%,51.9%	9.1% 6.3%,12.7%	2.4% 0.2%,4.9%	26.9% 17.4%,38.0%
25–44	SimSmoke	24.1%	20.5%	19.1%	20.7%				
	CCHS 95% CI	27.9% 26.0%,29.7%	21.4% 19.7%,23.1%	19.9% 17.7%,22.0%	28.7% 21.1%,36.6%	8.0% 0.4%,15.9%	1.3% 0.1%,2.7%	1.6% 0.4%,2.9%	0.2% -4.7%,5.7%
45–64	SimSmoke	18.4%	16.4%	15.9%	13.9%				
	CCHS 95% CI	25.1% 23.3%,27.0%	20.7% 19.2%,22.3%	17.6% 15.9%,19.4%	29.9% 22.7%,36.7%	16.0% 8.8%,22.8%	2.5% 1.3%,3.7%	1.2% 0.0%,2.4%	6.3% 1.7%,10.9%
65+	SimSmoke	8.2%	7.3%	7.1%	12.5%				
	CCHS 95% CI	9.9% 8.8%,11.1%	10.6% 9.3%,11.9%	9.2% 8.1%,10.3%	7.1% -4.0%,18.2%	-5.5% -16.6%,5.6%	-0.7% -2.2%,0.8%	-2.9% -4.9%,-0.8%	5.6% 0.2%,11.3%
Females									
18+	SimSmoke	13.9%	11.4%	10.8%	22.6%				
	CCHS 95% CI	17.6% 16.8%,18.5%	13.1% 12.4%,13.8%	10.3% 9.5%,11.0%	41.5% 37.5%,46.0%	18.8% 14.9%,23.4%	3.3% 2.5%,4.3%	1.5% 0.7%,2.4%	8.5% 5.5%,12.0%
18–24	SimSmoke	13.7%	10.6%	10.2%	25.0%				
	CCHS 95% CI	18.0% 15.2%,20.9%	10.2% 7.8%,12.7%	3.9% 2.3%,5.5%	78.3% 69.4%,87.2%	53.3% 44.4%,62.2%	13.9% 10.2%,19.1%	4.9% 1.5%,8.8%	36.5% 24.9%,50.9%
25–44	SimSmoke	15.8%	11.7%	10.6%	33.0%				
	CCHS 95% CI	19.9% 18.4%,21.4%	14.7% 13.5%,15.9%	11.3% 9.9%,12.8%	43.2% 35.7%,50.3%	10.2% 2.7%,17.3%	1.9% 0.5%,3.5%	0.1% -1.2%,1.4%	7.4% 1.8%,13.0%
45–64	SimSmoke	14.5%	12.4%	11.7%	19.3%				
	CCHS 95% CI	20.1% 18.6%,21.6%	15.7% 14.4%,17.0%	13.0% 11.6%,14.4%	35.3% 28.4%,42.3%	16.0% 9.0%,23.0%	2.7% 1.4%,4.0%	1.5% 0.2%,2.9%	6.0% 1.2%,11.0%
65+	SimSmoke	10.0%	9.9%	10.1%	-0.4%				
	CCHS 95% CI	8.9% 8.0%,9.8%	8.2% 7.2%,9.2%	7.1% 6.3%,7.9%	20.2% 11.2%,29.2%	20.6% 11.6%,29.6%	2.8% 1.5%,4.3%	1.2% -0.7%,3.3%	7.6% 2.5%,13.0%

1. Relative reductions are measured by the relative decrease within a certain period, e.g., prevalence reduces by 25% from 20% in 2012 to 15% in 2019

2. Differences between SimSmoke model projections and survey estimates are measured by the relative reductions within the specified time period

3. Annual reductions are the average annual relative reduction when transforming the relative reduction within the specified time period evenly to the between years, e.g., the annual reduction from 20% in 2012 to 15% in 2020 is 1-(15%/20%)^[1/(2020-2012)]^ = 3.5%

4. Annual differences are measured by the difference between the annual reduction in SimSmoke projections and survey estimates within the specified time period

From 2012 to 2020, CTUMS (Table 2) yielded an implied relative reduction in male aged 15+ smoking prevalence of 34.3%, which is 14.9% greater than the 19.3% reduction from No-NVP *SimSmoke,* implying NVP-related average annual relative gains of 2.5%, which was significant with 95% CI: 1.1–3.8. For females ages 15+, CTUMS yielded a 2.5% average annual NVP-related gain. CTUMS yielded higher average NVP-related post-TVPA annual gains of 6.8% for males and 10.3% for females. CCHS (Table 3) implied significant average annual relative gains in ages 18+ smoking prevalence of 2.4% for males and 3.3% for females. NVP-related post-TVPA annual relative gains were 5.8% for males and 8.5% for females.

For ages 15–24, CTUMS implied annual post-TVPA gains of 13.4% for males and 20.4% for females, and CCHS implied post-TVPA annual gains of 26.9% for males and 36.5% for females. For ages 25–44, males also showed relatively large post-NVP era gains using CTUMs, but females showed a large gain only in the post-TVA period. For ages 45–64, CTUMS and CCHS implied gains of 2.5% for males with substantially higher post-TVPA gains, but CTUMS only implied gains for females. For ages 65+, CTUMS implied annual losses for males, while CCHS implied annual gains for females.

### Impact of NVP use during the period 2012–2020 on prevalence and smoking-attributable deaths

Table 4 shows projected smoking prevalence and total smoking-attributable deaths and deaths averted from 2012 to 2060. Comparing CTUMS annual NVP-adjusted to No-NVP SADs, *SimSmoke* projected 71,878 male and 28,624 female SADs averted (which after summing projected estimates equals 100,501 in total). Using CCHS, *SimSmoke* projected 44,535 male and 54,259 female SADs averted (totaling 98,794) from 2012 to 2060. These estimates implied a 6.6–6.7% decrease in SADs in the NVP relative to the No-NVP scenario. While the impact of NVP use on smoking is greatest at younger ages, the impact on SADs is delayed and tends to occur at later ages; from 2012 to 2060, 17,127 SADs are averted among those ages 35–54 compared to 83,374 SADs averted by those ages 55+ based on CTUMS adjustments or 11,193 SADs for age 35–54 and 87,600 SADs for age 55+ based on CCHS adjustment.

**Table 4:** Projected smoking prevalence, smoking-attributable deaths, No-NVP and NVP-adjusted *Simsmoke*, males and females, 2012–2060

	Source of estimate	2012	2020	2040	2060	Percent change 2012–2020	Percent change 2012–2060
Prevalence for age 15+	No-NVP	18.3%	14.8%	10.3%	8.8%	-19.3%	-51.6%
	CTUMS-adjusted	18.3%	11.5%	9.0%	8.6%	-36.9%	-53.0%
	CCHS-adjusted	18.3%	13.0%	9.5%	8.7%	-29.0%	-52.6%
Female prevalence							
Prevalence for age 15+	No-NVP	13.7%	10.6%	7.2%	5.6%	-22.3%	-59.2%
	CTUMS-adjusted	13.7%	9.0%	6.5%	5.3%	-34.5%	-61.5%
	CCHS-adjusted	13.7%	8.4%	6.3%	5.2%	-38.3%	-61.7%
Male SADs and lives saved							
	Source of estimate	2012	2020	2040	2060	Cumulative 2012–2020	Cumulative 2012–2060
SADs	No-NVP	17,019	17,946	19,523	13,959	157,015	876,682
	CTUMS-adjusted	17,019	17,477	17,509	11,722	155,139	804,805
	Lives saved	0	468	2015	2237	1875	71,878
	CCHS-adjusted	17,019	17,620	18,287	12,679	155,650	832,147
	Lives saved	0	326	1237	1280	1365	44,535
Female SADs and lives saved							
SADs	No-NVP	10,042	11,047	14,658	10,367	95,382	612,558
	CTUMS-adjusted	10,042	10,643	13,991	9446	93,648	583,934
	Lives saved	0	404	667	920	1734	28,624
	CCHS-adjusted	10,042	10,160	13,281	9168	91,531	558,299
	Lives saved	0	887	1377	1199	3850	54,259

1. SADs = smoking-attributable deaths

2. CTUMS-adjusted refers to NVP-implied estimates to reflect the reductions in smoking prevalence using the CTUS/CTADS/CTNS annual adjustments and the CCHS-adjusted refers to the NVP-implied estimates to reflect the additional reductions in smoking prevalence using the CCHS annual adjustments

3. The model does not consider health impacts from NVP use

## Discussion

Using our indirect method (Levy et al., 2021a, b) for estimating the potential impact of NVP use on smoking prevalence and smoking-attributable deaths in Canada, we found that smoking prevalence declined more rapidly after 2012 when NVPs became more widely used. The accelerated decline in smoking was especially pronounced for younger adults where NVP use is more common. The decline in smoking prevalence is also more pronounced in the post-TVPA (2018–2020) compared to pre-TVPA (2017 and before) period, suggesting that increased accessibility to NVPs contributed to a reduction in Canadian smoking prevalence. The results, especially for young adults, are broadly consistent with other modeling studies (Levy et al., 2021a, b), including studies that explicitly modelled NVP transitions in the USA (Levy et al., 2021b; Mendez & Warner, 2021). In addition, empirical studies have found higher NVP use at younger ages (Bao et al., 2019; Levy et al., 2019b) and NVP-related reductions in youth and young adult smoking (Levy et al., 2019a; Meza et al., 2020). Since the declining trend in the uptake of cigarettes began before NVPs were available in the marketplace, we do not causally attribute the accelerated declines in smoking between 2012 and 2020 to NVP substitution. However, it is evident that the emergence of NVPs into the Canadian marketplace has not slowed the decline in smoking.

The findings on NVP-related reductions in Canadian smoking are similar to those found applying a similar method to other settings. In the USA, the inferred NVPs-related reduction in US adult smoking prevalence was of similar magnitude (15%) and was greater at younger ages (Levy et al., 2021b). However, much of the gain for Canada was estimated to occur after NVP regulations were relaxed in 2018 (thus expanding access to NVPs), whereas impacts were observed in the USA over the entire 2013–2018 period during which NVPs were largely unregulated. In England (Levy et al., 2021a), a 20% overall NVP-smoking reduction for adults was obtained, higher than for the USA and Canada, and more uniformly distributed over all ages. The greater impact may reflect the UK’s incorporation of NVPs in their national cessation treatment policy and strong cigarette-oriented policies, leading to greater incentive to switch from cigarettes to NVPs (National Centre for Smoking Cessation and Training (NCSCT), 2022).

We projected about 100,000 SADs averted from 2012 to 2060 as implied by 2012–2020 NVP-related smoking reductions in Canada. Any potential NVP-related reductions after 2020 are not included. We also did not include NVP-attributable deaths, although NVP-related mortality risks are generally considered substantially lower than cigarette smoking risks (McNeill et al., 2022; NASEM, 2018). Any impact of second-hand smoke exposure was also excluded.

These findings are subject to caveats. The implied NVP impacts rely on the validity of the model. *SimSmoke* has been validated across regions (Levy et al., 2008, 2012, 2014a, b, 2016a) with a wide variation in policies. *Canada SimSmoke* was calibrated for the pre-NVP era (1999–2012) and incorporated the impact of tobacco control policies. Smoking prevalence was reduced by 31% from cigarette-oriented policies implemented in the pre-NVP era, but only by 5% in the post-NVP era. Sensitivity analyses indicated that our implied impacts during the NVP era were insensitive to policy effect sizes over credible ranges (see Supplemental Report). Nevertheless, the indirectly inferred impact of NVPs implicitly assumes that access to NVPs is the only factor other than cigarette-oriented policies that would have influenced post-NVP smoking prevalence. Other factors may include COVID-related impacts, changes in industry behaviour, and changes in public attitudes toward tobacco.

Our indirect method also implicitly assumes that effect sizes of cigarette-oriented policies are the same in the NVP era as in the pre-NVP era. While NVPs may blunt the impact of some cigarette-oriented policies (e.g., through increased dual-use rather than quitting), it is also possible that NVPs may enhance policy impacts if smokers are more likely to substitute NVPs for cigarettes in response to stricter cigarette-oriented policies. Indeed, demand studies (Pesko et al., 2018; Zheng et al., 2017) indicate that NVPs are a substitute for cigarettes, and cessation studies (Beard et al., 2016; Levy et al., 2018b) indicate that NVPs are often used by those who are most heavily dependent (McNeill et al., 2019).

Another limitation of the model is that NVP-related impacts depend on the accuracy of survey estimates. Smoking prevalence estimates from CTUMS and CCHS varied considerably, especially for those ≥ age 65. Further study is merited on variations across the two surveys, and the impact of COVID-19 on smoking behaviours and changes in survey methodology (begun online only in 2020).

Finally, the projected survey trends may depend on how the post-NVP period is defined. Using CTUMS, the implied NVP-related reductions for those ages 15+ were 18.3% for males and 1.2% for females in 2012–2019 compared to 14.9% for males and 14.5% for females in 2012–2020. Using CCHS, the implied NVP-related reductions for those aged 18+ were 8.6% for males and 9.4% for females in 2012–2019 compared to 14.3% for males and 18.8% for females in 2012–2020. The generally greater impact using the 2020 end-date may reflect the longer period in which NVP restrictions were relaxed or the impact of COVID-19 (Gravely et al., 2021). However, the analysis also does not consider the impact of provincial NVP flavour bans implemented as early as April 2020 (Smoke-free Canada, 2022). Further study is merited on the impact of variation in the end date of the study as well as the impact of cigarette- and NVP-oriented policies.

## Conclusion

The emergence of nicotine vaping products into the Canadian marketplace, particularly legal access to NVPs in retail stores beginning in 2018, has not slowed the decline in smoking. Implied NVP-related reductions in smoking prevalence were most pronounced among younger smokers who are also more likely to use NVPs and especially in the post-TVPA period. Historical trends and ongoing cigarette-oriented tobacco control policies in Canada explain little of the accelerated reduction in smoking prevalence after NVPs became legally regulated and use became more prevalent. Our study indicates the potential public health impact of NVPs through reduced cigarette use, particularly among those at younger ages. However, trends in NVP and cigarette use and the impact of new policies on these trends should be carefully monitored. While these findings suggest a net positive impact for increased NVP access in reducing overall smoking prevalence, further research is needed to evaluate the explicit impact of NVP use on smoking initiation and cessation and on health outcomes.

## Contributions to knowledge

What does this study add to existing knowledge?

- The Canada *SimSmoke* simulation model is used to estimate trends in smoking prevalence controlling for prior trends and changes in cigarette-oriented policies, but does not explicitly incorporate the impact of nicotine vaping products (NVPs).
- The No-NVP counterfactual projections are compared to actual smoking rates to estimate the implicit net impact of NVPs.
- The analysis shows that smoking prevalence declined at a more rapid rate than projected by the model during the period when NVPs became more common.
- Smoking prevalence in Canada declined most, particularly among young adults, when NVP sales were legalized.

What are the key implications for public health interventions, practice or policy?

- The analysis suggests that NVP use may provide important public health benefits in terms of reducing cigarette use and smoking-attributable deaths, but further analysis is necessary to monitor cigarette use and its relationship to NVP use.

### Supplementary Information

ESM 1 (DOCX 113 kb)

## Data Availability

All data will be made available upon request.

## Code availability

All code will be made available upon request.
