# Supplementary material for: Comparison of smoking prevalence in Canada before and after nicotine vaping product access using the SimSmoke model
Source: Can J Public Health. 2023 Aug 4;114(6):992–1005. doi: 10.17269/s41997-023-00792-3 (PMC10661672; doi:10.17269/s41997-023-00792-3)
Supplement: Supplementary file 1 — (DOCX 113 kb) [file 41997_2023_792_MOESM1_ESM.docx]

**Supplement 1: Timeline of Canada Tobacco Control Policies**


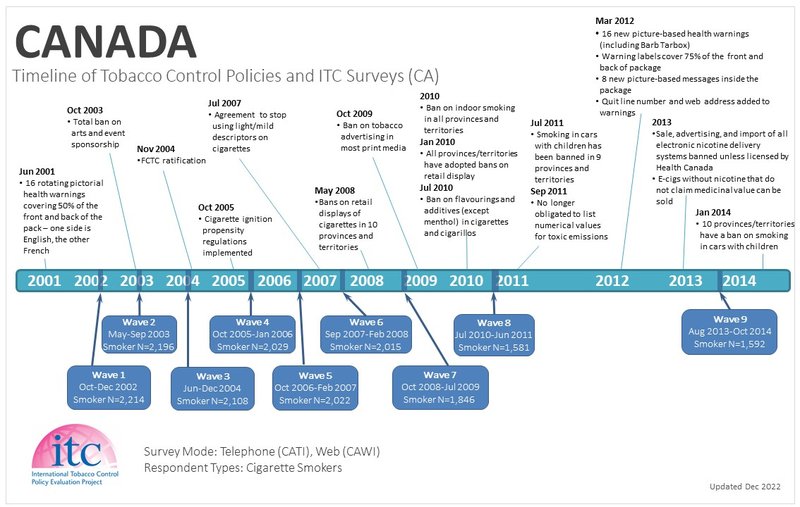


**Supplement 2: Table 1. Validation of SimSmoke Smoking Prevalence vs. Canadian Surveys, by Age and Sex, 1999-2012**

| **Male** | | | | | | | | | | |
| --- | --- | --- | --- | --- | --- | --- | --- | --- | --- | --- |
| **Ages** | **Sources** | **1999** | **2001** | **2006** | **2012** | **Relative reduction 1999-2006** | **Relative reduction 2001-2006** | **Relative reduction 2006-2012** | **Relative reduction 1999-2012** | **Relative reduction 2001-2012** |
| 15+ | SimSmoke | 27.0% | 26.3% | 21.0% | 18.3% | -22.2% | -20.1% | -13.0% | -32.3% | -30.4% |
|  | CTUMS | 26.9% | 23.3% | 20.2% | 17.8% | -13.4% |  | -11.9% | -33.9% |  |
|  | 95% CI | 26.2%,27.0% | 22.9%,23.7% | 19.8%,20.6% | 17.4%,18.2% |  |  |  |  |  |
| 18+ | SimSmoke | 27.5% | 26.9% | 21.7% | 18.8% | -21.3% | -19.5% | -13.2% | -31.7% | -30.1% |
|  | CCHS |  | 29.3% | 24.8% | 23.8% |  | -15.4% |  |  | -18.8% |
|  | 95% CI |  | 28.7%,29.9% | 24.3%,25.4% | 22.8%,24.8% |  |  |  |  |  |
| 15-24 | SimSmoke | 31.0% | 29.1% | 18.4% | 16.0% | -40.9% | -36.9% | -12.8% | -48.5% | -45.0% |
|  | CTUMS | 31.4% | 26.7% | 21.3% | 15.7% | -15.0% |  | -26.3% | -49.9% |  |
|  | 95% CI | 30.5%,31.5% | 26.2%,27.2% | 20.8%,21.8% | 15.3%,16.2% |  |  |  |  |  |
| 18-24 | SimSmoke | 36.7% | 34.9% | 22.2% | 19.0% | -39.4% | -36.3% | -14.3% | -48.0% | -45.4% |
|  | CCHS |  | 34.2% | 28.7% | 27.2% |  | -16.1% |  |  | -20.5% |
|  | 95% CI |  | 32.2%,36.3% | 27.0%,30.4% | 24.0%,30.3% |  |  |  |  |  |
| 25-44 | SimSmoke | 32.4% | 32.5% | 27.3% | 24.1% | -15.7% | -16.0% | -11.7% | -25.5% | -25.8% |
|  | CTUMS | 32.5% | 27.4% | 23.8% | 22.9% | -15.7% |  | -3.8% | -29.6% |  |
|  | 95% CI | 31.3%,32.7% | 26.7%,28.1% | 23.1%,24.6% | 22.0%,23.8% |  |  |  |  |  |
|  | CCHS |  | 34.3% | 30.3% | 27.9% |  | -11.7% |  |  | -18.7% |
|  | 95% CI |  | 33.3%,35.2% | 29.3%,31.2% | 26.0%,29.7% |  |  |  |  |  |
| 45-64 | SimSmoke | 23.3% | 22.8% | 20.6% | 18.4% | -11.3% | -9.5% | -10.6% | -20.7% | -19.1% |
|  | CTUMS | 22.6% | 21.5% | 21.2% | 18.1% | -4.9% |  | -14.6% | -20.0% |  |
|  | 95% CI | 21.7%,23.1% | 20.8%,22.1% | 20.5%,21.9% | 17.4%,18.8% |  |  |  |  |  |
|  | CCHS |  | 27.7% | 23.2% | 25.1% |  | -16.2% |  |  | -9.4% |
|  | 95% CI |  | 26.8%,28.7% | 22.2%,24.1% | 23.3%,27.0% |  |  |  |  |  |
| 65+ | SimSmoke | 13.6% | 12.5% | 9.2% | 8.2% | -32.8% | -26.4% | -10.9% | -40.1% | -34.5% |
|  | CTUMS | 13.0% | 10.6% | 7.1% | 8.4% | -18.5% |  | 18.3% | -35.4% |  |
|  | 95% CI | 12.0%,13.7% | 9.7%,11.5% | 6.5%,7.8% | 7.7%,9.1% |  |  |  |  |  |
|  | CCHS |  | 13.2% | 11.0% | 9.9% |  | -16.7% |  |  | -25.0% |
|  | 95% CI |  | 12.1%,14.2% | 10.2%,11.9% | 8.8%,11.1% |  |  |  |  |  |

| **Female** | | | | | | | | | | |
| --- | --- | --- | --- | --- | --- | --- | --- | --- | --- | --- |
| **Ages** | **Sources** | **1999** | **2001** | **2006** | **2012** | **Relative reduction 1999-2006** | **Relative reduction 2001-2006** | **Relative reduction 2006-2012** | **Relative reduction 1999-2012** | **Relative reduction 2001-2012** |
| 15+ | SimSmoke | 22.5% | 21.2% | 16.3% | 13.7% | -27.3% | -23.0% | -16.1% | -39.0% | -35.4% |
|  | CTUMS | 22.7% | 19.2% | 16.4% | 13.3% |  | -14.6% | -18.9% | -41.5% | -30.7% |
|  | 95% CI | 22.2%,22.9% | 18.9%,19.4% | 16.0%,16.7% | 13.1%,13.6% |  |  |  |  |  |
| 18+ | SimSmoke | 22.6% | 21.4% | 16.7% | 13.9% | -26.2% | -22.3% | -16.4% | -38.3% | -35.0% |
|  | CCHS |  | 24.0% | 20.1% | 17.6% |  | -16.3% |  |  | -26.7% |
|  | 95% CI |  | 23.4%,24.5% | 19.6%,20.6% | 16.8%,18.5% |  |  |  |  |  |
| 15-24 | SimSmoke | 27.2% | 24.3% | 14.5% | 12.1% | -46.7% | -40.2% | -16.5% | -55.5% | -50.1% |
|  | CTUMS | 27.1% | 24.5% | 17.1% | 11.8% |  | -30.2% | -31.0% | -56.4% | -51.8% |
|  | 95% CI | 26.3%,27.2% | 24.1%,24.9% | 16.7%,17.5% | 11.4%,12.2% |  |  |  |  |  |
| 18-24 | SimSmoke | 30.2% | 27.7% | 16.7% | 13.7% | -44.8% | -39.8% | -18.0% | -54.8% | -50.6% |
|  | CCHS |  | 30.6% | 23.7% | 18.0% |  | -22.5% |  |  | -41.2% |
|  | 95% CI |  | 29.0%,32.2% | 22.2%,25.2% | 15.2%,20.9% |  |  |  |  |  |
| 25,44 | SimSmoke | 27.0% | 25.9% | 20.3% | 15.8% | -24.7% | -21.6% | -22.3% | -41.5% | -39.1% |
|  | CTUMS | 27.6% | 21.6% | 20.3% | 15.2% |  | -6.0% | -25.1% | -44.9% | -29.6% |
|  | 95% CI | 26.7%,28.0% | 21.1%,22.2% | 19.6%,21.1% | 14.6%,15.9% |  |  |  |  |  |
|  | CCHS |  | 28.1% | 23.2% | 19.9% |  | -17.4% |  |  | -29.2% |
|  | 95% CI |  | 27.3%,29.0% | 22.5%,24.0% | 18.4%,21.4% |  |  |  |  |  |
| 45-64 | SimSmoke | 20.6% | 19.8% | 16.7% | 14.5% | -18.8% | -15.5% | -13.3% | -29.6% | -26.8% |
|  | CTUMS | 20.5% | 17.7% | 16.1% | 14.4% |  | -9.0% | -10.6% | -29.9% | -18.6% |
|  | 95% CI | 19.8%,21.1% | 17.1%,18.3% | 15.6%,16.7% | 13.9%,15.0% |  |  |  |  |  |
|  | CCHS |  | 23.2% | 20.7% | 20.1% |  | -10.8% |  |  | -13.4% |
|  | 95% CI |  | 22.3%,24.1% | 19.8%,21.6% | 18.6%,21.6% |  |  |  |  |  |
| 65+ | SimSmoke | 11.1% | 10.7% | 9.4% | 10.0% | -27.3% | -23.0% | -16.1% | -39.0% | -35.4% |
|  | CTUMS | 10.7% | 10.8% | 8.3% | 9.3% |  | -14.6% | -18.9% | -41.5% | -30.7% |
|  | 95% CI | 9.9%,11.4% | 10.1%,11.4% | 7.8%,8.9% | 8.8%,9.8% |  |  |  |  |  |
|  | CCHS |  | 11.1% | 9.8% | 8.9% | -26.2% | -22.3% | -16.4% | -38.3% | -35.0% |
|  | 95% CI |  | 10.4%,11.9% | 9.1%,10.5% | 8.0%,9.8% |  |  |  |  |  |

Notes: Relative reductions are measured by the relative decrease within certain period, i.e., [(year 2-year 1)/year 1].
